# Supplementary material for: T-DOpE probes reveal sensitivity of hippocampal oscillations to cannabinoids in behaving mice
Source: Nat Commun. 2024 Feb 24;15:1686. doi: 10.1038/s41467-024-46021-4 (PMC10894268; doi:10.1038/s41467-024-46021-4)
Supplement: Supplementary file 1 — Supplementary information [file 41467_2024_46021_MOESM1_ESM.pdf]

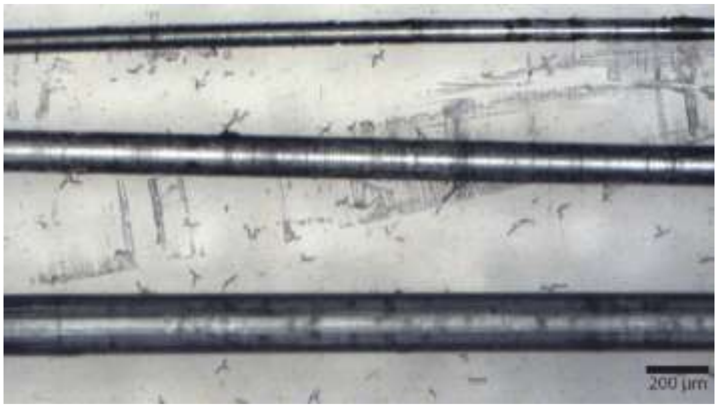

Supplementary Figure 1: Microscope image of three various thickness of fiber achieved with thermal tapering.

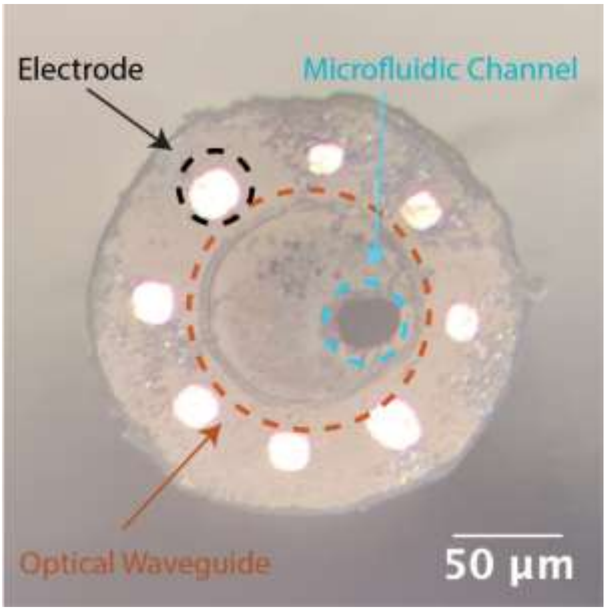

Supplementary Figure 2: Cross section of a probe design with 20 µm microfluidic channel.

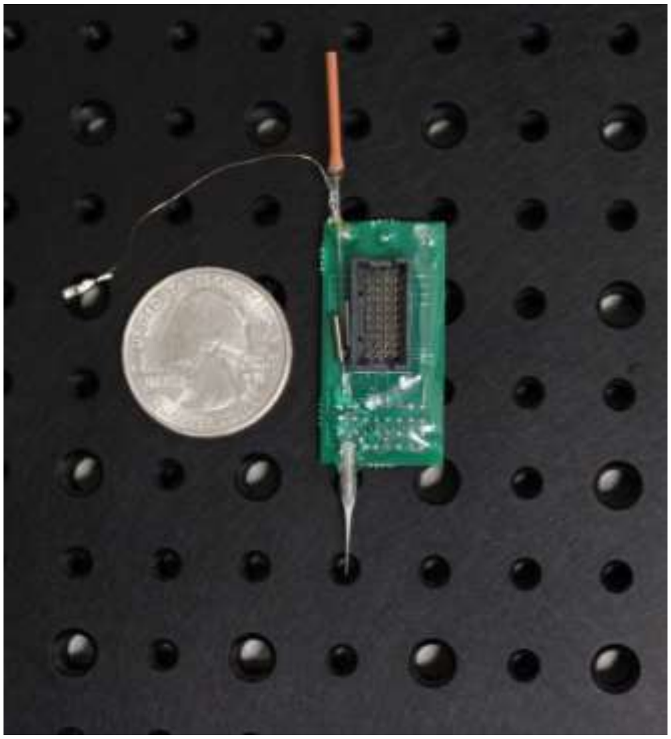

Supplementary Figure 3: Photograph of T-DOpE probe connected to PCB, ferrules, and microfluidic tube. T-DOpE probe connected to PCB was mainly used to monitor and manipulate CA1 circuitry acutely in behaving mice.

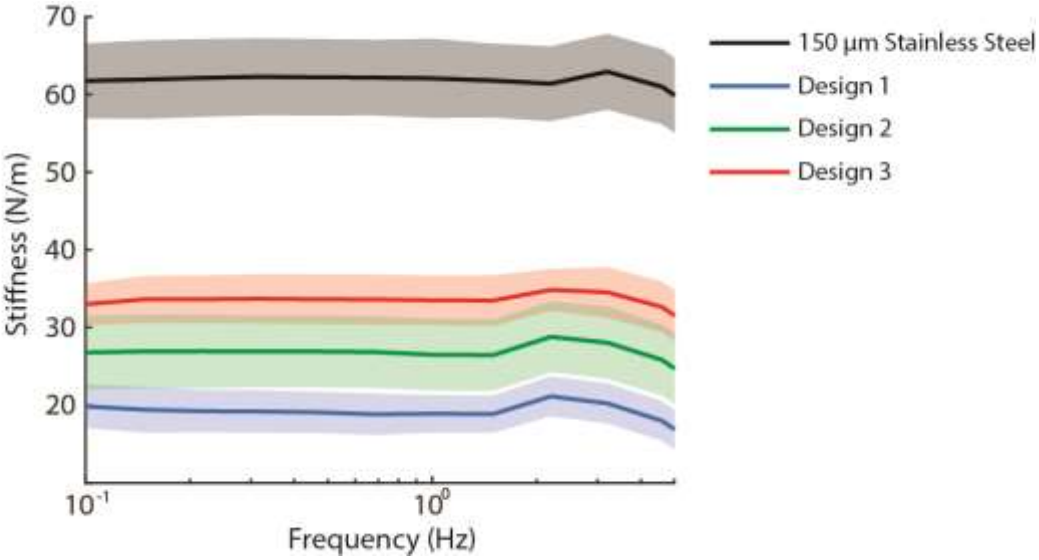

Supplementary Figure 4: Stiffness vs Frequency measurement using a dynamic mechanical analyzer. (n=4 for each designs)

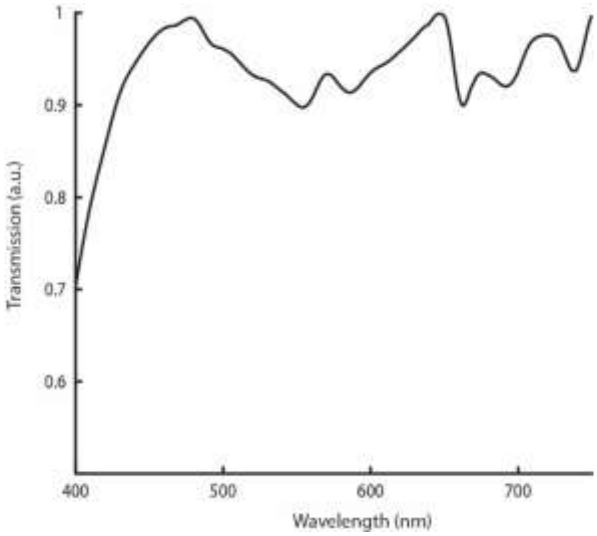

Supplementary Figure 5: Transmission Spectrum of the optical waveguide

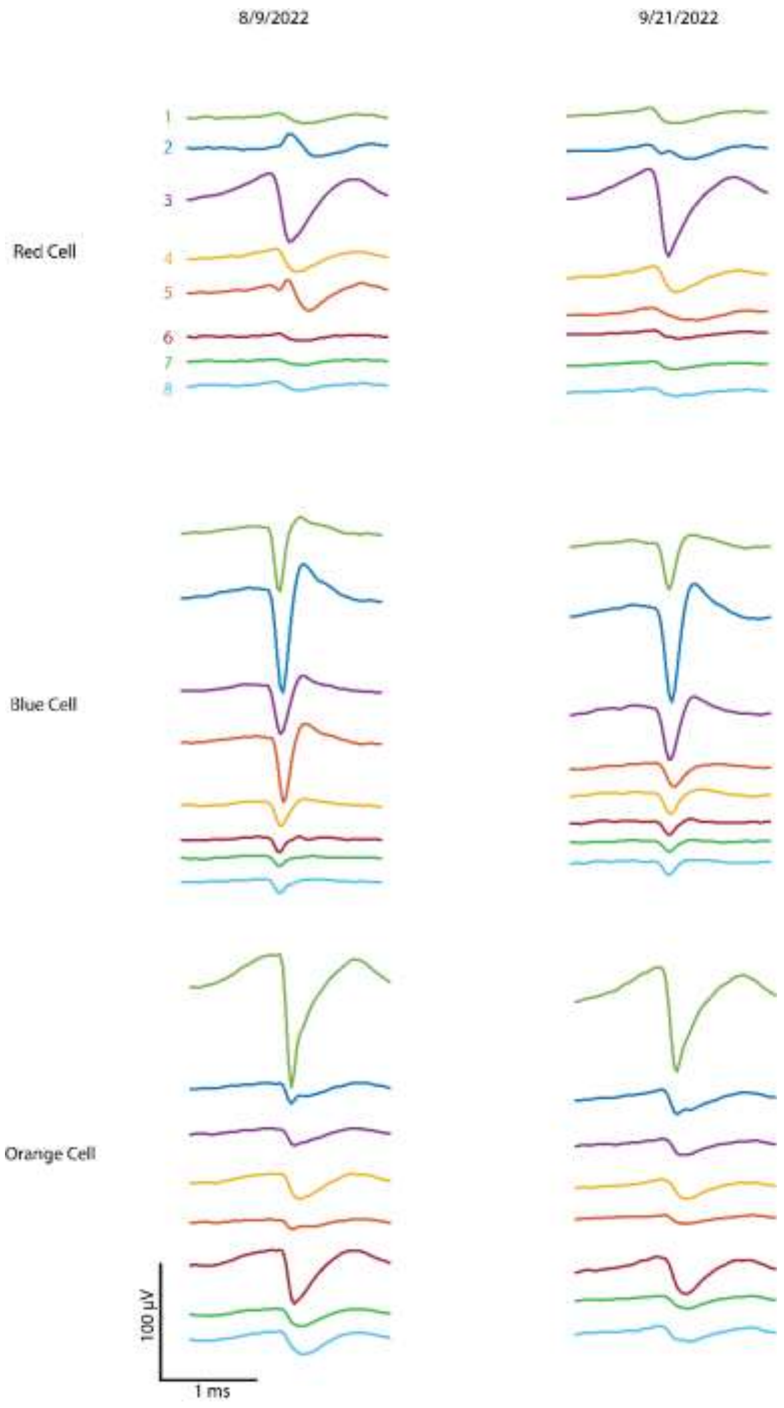

Supplementary Figure 6: Average waveform of identified cells in fig. 4c from each electrode. Red and blue cell maintained its monosynaptic connection over 43 days. The electrode number is color-coded and is not correlated with the location of the recording site.

Treatment Group	Mouse ID	Session ID	# of Units	Putative Cell type	Isolation Distance	% ISI Violation
Saline	m449	m449_230921	3 units	PYR	26.3	0.12
				INT	21.0	0.09
				INT	27.6	0.01
	m450	m450_230921	1 unit	INT	28.2	0.27
	m451	m451_230922	1 unit	INT	38.4	0.03
	m452	m452_290922	1 unit	PYR	19.0	3.31
Drug Vehicle	m400	m400_221219	MUA only			
	m401	m401_221224	MUA only			
	m402	m402_221209	5 units	PYR	18.7	0.51
				PYR	25.9	0.21
				PYR	26.6	2.04
				PYR	14.4	0.72
				INT	28.7	0.24
		m402_221212	2 units	PYR	23.8	0.30
				INT	22.9	1.45
	m441	m441_231002	1 unit	PYR	21.5	2.16
	m442	m442_231006	1 unit	PYR	18.7	0.15
CP-55,940	m402	m402_220926	3 units	PYR	44.1	0.01
				PYR	4.9	0.62
				INT	1.01	0.95
	m403	m403_221011	3 units	PYR	33.2	0.21
				INT	35.0	1.82
				INT	38.7	0.47
	m404	m404_221018	MUA only			
	m441	m441_231005	MUA only			
	m442	m442_231010	7 units	PYR	30.8	0.41
				PYR	26.2	0.12
				PYR	31.6	0.01
				PYR	30.4	0.36
				PYR	23.1	0.46
				INT	40.8	0.01
				INT	41.5	0.06
CP-55,940 & ChR2+	m406	m406_230201	3 units	PYR	27.1	1.44
				INT	29.9	0.18
				INT	31.4	0.11
	m432	m432_231019	MUA only			
	m433	m433_231013	7 units	PYR	21.8	0.18
				PYR	25.9	0.18
				PYR	17.5	0.31
				PYR	21.5	0.99
				INT	22.4	0.12
				INT	36.1	0.05
				INT	42.4	0.25
	m435	m435_231016	MUA only			

Supplementary Table 1: Sorted units and their cluster qualities over all acute sessions

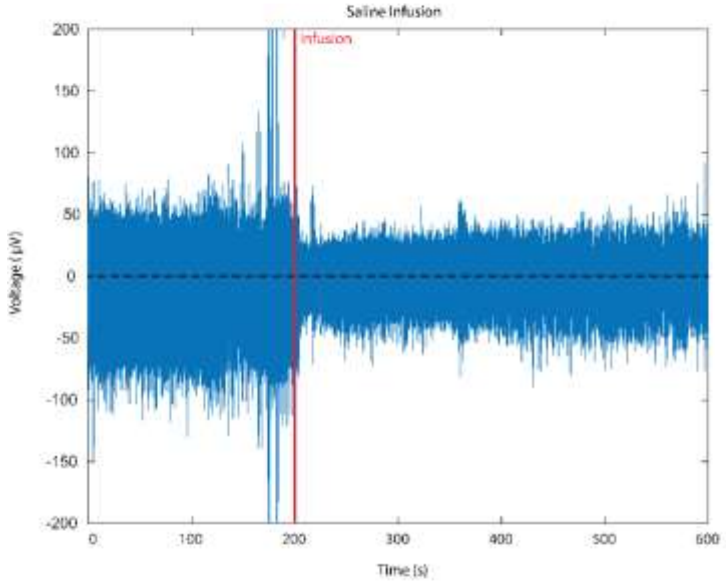
Saline Infusion
Infusion
Voltage (μV)
Time (s)

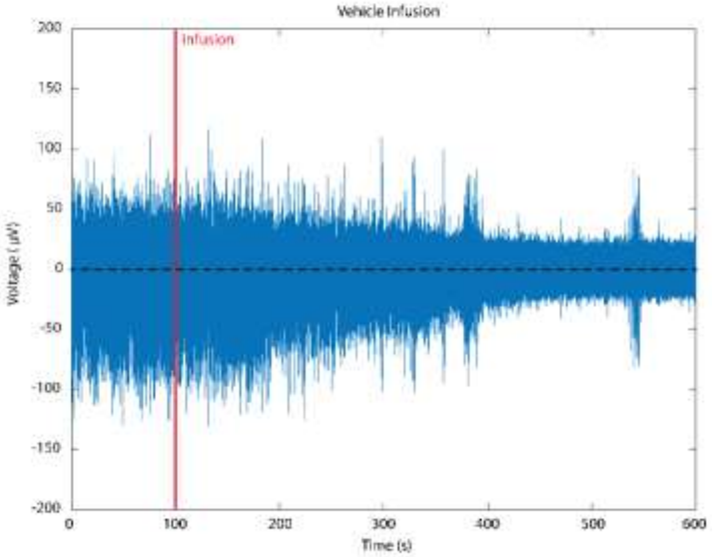
Vehicle Infusion
Infusion
Voltage (μV)
Time (s)

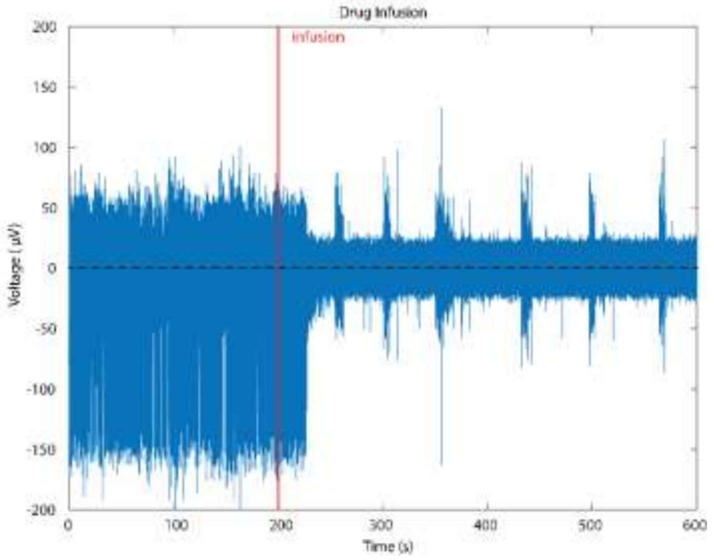
Drug Infusion
Infusion
Voltage (μV)
Time (s)

Supplementary Figure 7: 10-minute single-unit resolution extracellular trace (600- 8000Hz) where the cells are inevitably displaced due to infusion (200nL, $1nLs^{-1}$). Before infusion, the multi-unit activities were recorded with T-DOpE probe. For all infusion (saline, vehicle, and drug), the neurons are pushed away, and the action potentials of these neurons cannot be recorded until the diffusion has finished. The symmetric noise are the muscle artifacts from the mice.

Minmax normalized average speed pre and post infusion (1 hour each)

Infusion type	Mouse ID	Mouse Session	Minmax norm. avg. speed & std preinfusion	Minmax norm. avg. speed & std postinfusion	P-value for paired t-test	% time spent running preinfusion (%)	% time spent running postinfusion (%)	P-value for paired t-test
Drug Vehicle	m400	m400_221219	0.0473;0.0825	0.0532; 0.0960	0.9474 (Avg.Speed pre and post infusion is not stastically significant)	8.26	10.78	0.3353 (%time running pre and postinfusion is not stastically significant)
	m401	m401_221224	0.0698;0.1257	0.0964; 0.1688		13.82	12.57	
	m402	m402_221212	0.0825;0.1535	0.0901; 0.1485		20.55	13.82	
	m442	m442_231006	0.0500;0.1199	0.0418; 0.1240		9.76	6.33	
CP-55,940	m402	m402_220926	0.0844;0.1739	0.0901; 0.1360	0.8293 (Avg.Speed pre and post infusion is not stastically significant)	17.10	12.31	0.8277 (%time running pre and post infusion is not stastically significant)
	m403	m403_221011	0.0351;0.0918	0.0455; 0.1010		6.89	7.40	
	m404	m404_221018	0.0633;0.1149	0.0943; 0.1543		11.92	20.11	
	m441	m441_231005	0.0737;0.1943	0.0723; 0.1736		11.76	12.51	
	m442	m442_231010	0.0658;0.1609	0.0606; 0.1607		13.20	11.16	

Supplementary Table 2: Average speed and time spent running of the mice pre and post infusion. Paired two-sided t-tests were used for the comparison.

Linear mixed estimate model fitted by using fitlme (Matlab)
Power of theta as function of intercept and running speed grouped by infusion conditions

Infusion type	Mouse ID	Mouse Session	P-value for slopes	P-value for intercept
Drug Vehicle	m400	m400_221219	0.56272	8.454e-66
	m401	m401_221224	0.53787	1.4772e-34
	m402	m402_221212	0.19012	1.4671e-105
	m442	m442_231006	0.629	6.1918e-32
CP-55,940	m402	m402_220926	0.37642	3.176e-104
	m403	m403_221011	0.05195	1.5538e-58
	m404	m404_221018	0.69183	4.0685e-109
	m441	m441_231005	0.10348	1.0033e-38
	m442	m442_231010	0.39025	1.6338e-24

Supplementary Table 3: Linear mixed-estimate model of the theta power, velocity of the mice, and the infusion condition.

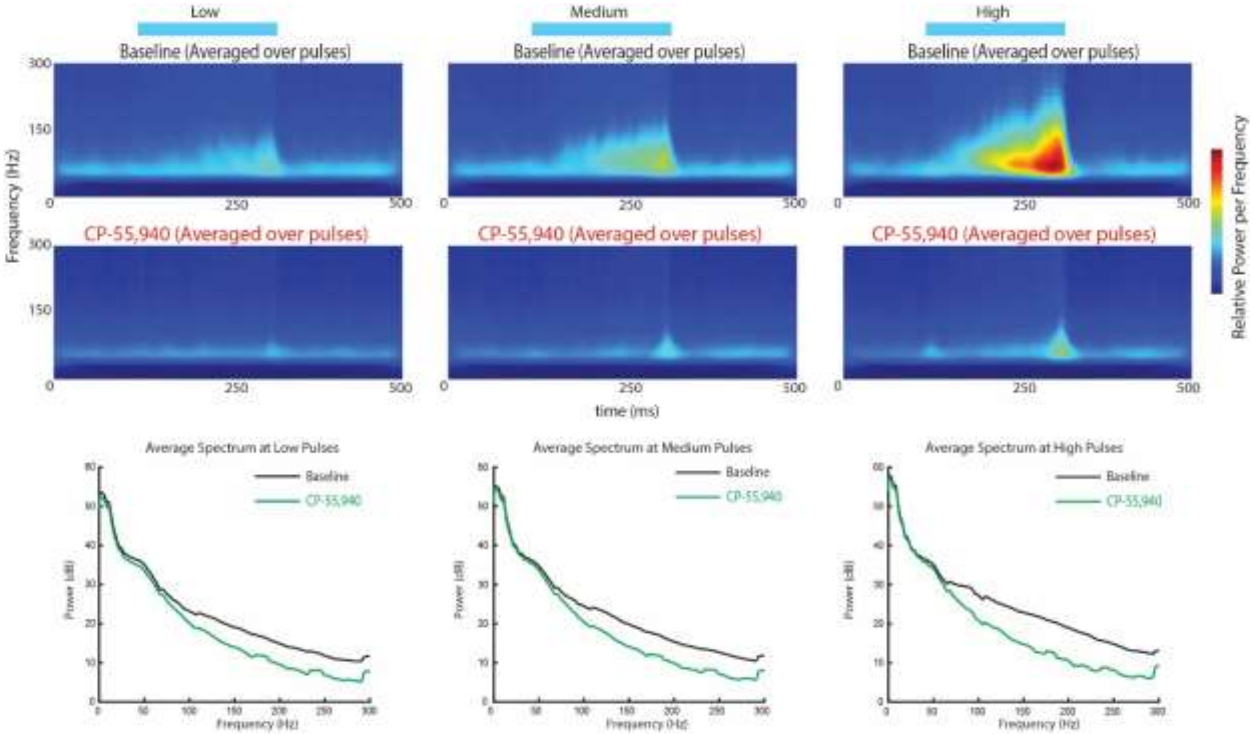

Supplementary Figure 8: Wavelet transforms and spectrum at low medium and high optical pulses.
