# Supplementary material for: T-DOpE probes reveal sensitivity of hippocampal oscillations to cannabinoids in behaving mice
Source: Nat Commun. 2024 Feb 24;15:1686. doi: 10.1038/s41467-024-46021-4 (PMC10894268; doi:10.1038/s41467-024-46021-4)
Supplement: Supplementary file 3 — Description of Additional Supplementary Information [file 41467_2024_46021_MOESM3_ESM.pdf]

Description of Additional Supplementary Files

File Name: Supplementary Video 1

Description: The probe bending to 90º with a radius of curvature of 1.07 cm without breaking to demonstrate the flexibility of the probe.

File Name: Supplementary Video 2

Description: Video of the probe in a 0.6% agarose gel to demonstrate the drug infusion. The inserted probe demonstrates the infusion of three different food dyes at three different heights in the phantom. (12x speed)
