# Supplementary material for: T-DOpE probes reveal sensitivity of hippocampal oscillations to cannabinoids in behaving mice
Source: Nat Commun. 2024 Feb 24;15:1686. doi: 10.1038/s41467-024-46021-4 (PMC10894268; doi:10.1038/s41467-024-46021-4)
Supplement: Supplementary file 7 — Reporting Summary [file 41467_2024_46021_MOESM7_ESM.pdf]

nature portfolio

Corresponding author(s): Xiaoting Jia

Last updated by author(s): Jan 25, 2024

Reporting Summary

Nature Portfolio wishes to improve the reproducibility of the work that we publish. This form provides structure for consistency and transparency in reporting. For further information on Nature Portfolio policies, see our Editorial Policies and the Editorial Policy Checklist.

Statistics

For all statistical analyses, confirm that the following items are present in the figure legend, table legend, main text, or Methods section.

n/a	Confirmed	
☐	☒	The exact sample size (*n*) for each experimental group/condition, given as a discrete number and unit of measurement
☐	☒	A statement on whether measurements were taken from distinct samples or whether the same sample was measured repeatedly
☐	☒	The statistical test(s) used AND whether they are one- or two-sided<br>*Only common tests should be described solely by name; describe more complex techniques in the Methods section.*
☐	☒	A description of all covariates tested
☐	☒	A description of any assumptions or corrections, such as tests of normality and adjustment for multiple comparisons
☐	☒	A full description of the statistical parameters including central tendency (e.g. means) or other basic estimates (e.g. regression coefficient) AND variation (e.g. standard deviation) or associated estimates of uncertainty (e.g. confidence intervals)
☐	☒	For null hypothesis testing, the test statistic (e.g. *F*, *t*, *r*) with confidence intervals, effect sizes, degrees of freedom and *P* value noted<br>*Give P values as exact values whenever suitable.*
☒	☐	For Bayesian analysis, information on the choice of priors and Markov chain Monte Carlo settings
☒	☐	For hierarchical and complex designs, identification of the appropriate level for tests and full reporting of outcomes
☒	☐	Estimates of effect sizes (e.g. Cohen's *d*, Pearson's *r*), indicating how they were calculated

Our web collection on statistics for biologists contains articles on many of the points above.

Software and code

Policy information about availability of computer code

Data collection	All the software used in the data collection has been described in methods section of manuscript. Intan Software was used to collect data and Spike2 Ver 9.03 combined with CED to stimulate optogenetic experiments.
Data analysis	A combination of software associated with hardware, repositories publicly available on Github (Buzcode, Kilosort, Cell Explorer and Phy) , and custom matlab code was used. Custom Matlab (Mathworks, Confirmed in version 2021B and 2018A) code available upon request to authors.

For manuscripts utilizing custom algorithms or software that are central to the research but not yet described in published literature, software must be made available to editors and reviewers. We strongly encourage code deposition in a community repository (e.g. GitHub). See the Nature Portfolio guidelines for submitting code & software for further information.

Data

Policy information about availability of data

All manuscripts must include a data availability statement. This statement should provide the following information, where applicable:

- Accession codes, unique identifiers, or web links for publicly available datasets
- A description of any restrictions on data availability
- For clinical datasets or third party data, please ensure that the statement adheres to our policy

All data published in this study available upon reasonable request. Additionally, all custom code available upon reasonable request.

Research involving human participants, their data, or biological material

Policy information about studies with human participants or human data. See also policy information about sex, gender (identity/presentation), and sexual orientation and race, ethnicity and racism.

Reporting on sex and gender	n/a
Reporting on race, ethnicity, or other socially relevant groupings	n/a
Population characteristics	n/a
Recruitment	n/a
Ethics oversight	n/a

Note that full information on the approval of the study protocol must also be provided in the manuscript.

Field-specific reporting

Please select the one below that is the best fit for your research. If you are not sure, read the appropriate sections before making your selection.

☒ Life sciences ☐ Behavioural & social sciences ☐ Ecological, evolutionary & environmental sciences

For a reference copy of the document with all sections, see nature.com/documents/nr-reporting-summary-flat.pdf

Life sciences study design

All studies must disclose on these points even when the disclosure is negative.

Sample size	Data from 17 mice were reported in this study, 2 Chronic mice, 15 acute mice. Given the nature of these experiments, animal numbers were sufficient because of repeated drug or vehicle treatments, as well as number of stimulations (n=1200 in each condition).
Data exclusions	Exclusion of data is described in manuscript. Only periods of high electrical noise, irrelevant to biological findings, were excluded from spiking and spectral analyses.
Replication	A total of 23 probes were used across experiments with no variability in performance (5 devices used for probe characterization, 18 were used in animal experiments.) Data from 17 mice were reported in this study (2 Chronic mice, 15 acute mice)
Randomization	Optical stimulation times were randomized.
Blinding	Experimental controls were conducted within animal for drug and optical delivery, blinding was irrelevant to experimental design.

Reporting for specific materials, systems and methods

We require information from authors about some types of materials, experimental systems and methods used in many studies. Here, indicate whether each material, system or method listed is relevant to your study. If you are not sure if a list item applies to your research, read the appropriate section before selecting a response.

Materials & experimental systems

n/a	Involved in the study	
☒	☐	Antibodies
☒	☐	Eukaryotic cell lines
☒	☐	Palaeontology and archaeology
☐	☒	Animals and other organisms
☒	☐	Clinical data
☒	☐	Dual use research of concern
☒	☐	Plants

Methods

n/a	Involved in the study	
☒	☐	ChIP-seq
☒	☐	Flow cytometry
☒	☐	MRI-based neuroimaging

Animals and other research organisms

Policy information about studies involving animals; ARRIVE guidelines recommended for reporting animal research, and Sex and Gender in Research

Laboratory animals	Mouse, Crossbred C57BL/6J (Jax #000664) x FVB/NJ (Jax #001800)
Wild animals	Did not involve
Reporting on sex	Mixed sex of animals were used across experiments. Groups within each sex were not large enough to conduct sufficiently powered statistical tests exploring sex differences. Previous studies exploring spikes, theta, sharp wave-ripples, and their respective interactions with synthetic cannabinoids, no sex dependent findings have been reported or are expected.
Field-collected samples	Did not involve
Ethics oversight	All animal procedures were approved by Virginia Tech Institutional Animal Care and Use Committee and Institutional Biosafety Committee and were carried out in accordance with National Institutes of Health Guide for the Care and Use of Laboratory Animals.

Note that full information on the approval of the study protocol must also be provided in the manuscript.
